# Older Adults’ Advance Aging and Life Satisfaction Levels: Effects of Lifestyles and Health Capabilities

**DOI:** 10.3390/bs13040293

**Published:** 2023-03-29

**Authors:** Dongwook Cho, Wookwang Cheon

**Affiliations:** College of Physical Education, Keimyung University, Daegu 42601, Republic of Korea; dcho@kmu.ac.kr

**Keywords:** advance aging, aging, functional assessment, gerotranscendence, health behavior, life satisfaction, lifestyle, older adults, vital sign

## Abstract

Many studies in the literature have examined older adults’ past and current lifestyles in either positive or negative association with their life satisfaction levels. Health capabilities naturally decline with aging and can consequently be related to older adults’ life satisfaction levels. Thus, the present study sought to examine the effects of age difference, lifestyles, and health capabilities on older adults’ life satisfaction levels. A total of 290 older adults from three clinical research centers in the United States completed a self-administered questionnaire on their lifestyles and life satisfaction levels, and their health capability assessments were evaluated. There was a significant effect of advancing age on life satisfaction levels among older adults. Additionally, engagement in exercise or physical activity significantly influenced life satisfaction levels. However, there were no statistical effects of vital signs and functional assessments of health capabilities on life satisfaction among older adults. The findings suggest that advancing age itself is the strongest factor in older adults’ life satisfaction. Additionally, engagement in exercise and physical activity can enhance life satisfaction levels as a supplemental factor among older adults. These findings can be beneficial to optimize life satisfaction levels through appropriate programs to encourage positive lifestyles among older adults.

## 1. Introduction

Aging is a natural process common to all individuals that presents as a decline of the functioning of major biological systems [1,2]. According to the Population Reference Bureau [3], 9 percent of the world population is 65 years or older, and this number is projected to increase to 16 percent of the world population by 2050. Previous research, however, suggests that defining the aging population as 65 and older might be a limited approach, as each individual has different health capabilities and lifestyles that affect their health age. Individuals also have a different perception of aging as they find themselves getting older [4].

Many studies have examined the individual’s aging process through different factors, including demographic characteristics, lifestyles, health conditions, social support, and perception of aging [1,4,5,6]. More specifically, previous studies in the literature discussed the effects of psychological well-being, happiness, and quality of life on an individual’s aging process [1,6]. For instance, Diener and Chan [7] systematically reviewed seven types of evidence that higher life satisfaction levels are positively associated with longevity and life expectancy among older adults. Another study also indicated that dissatisfaction levels were linearly associated with an increase in mortality through 20 years of a prospective cohort study [8]. Furthermore, it is reported that the decline of function accelerates with aging, which affects older adults’ psychological well-being and life satisfaction [9].

Life satisfaction is a complex concept that can be regarded as subjective well-being, quality of life, or happiness, as well as an overall positive assessment of an overarching criterion or ultimate outcome of human experience [10,11]. Currently, the population explosion of older adults requires attention concerning the importance of life satisfaction in a healthy life. Mannell and Snelgrove [12] stated that individuals are more likely to have better life satisfaction levels when their experiences match or exceed their expectations.

Older adults’ past or current lifestyles influence their life satisfaction level. An individual might base their standard of life satisfaction on their past health practices, including special diets, engagement in exercise, tobacco use, or alcohol consumption [13,14]. Previous studies examined participation in physical activity, exercise, or leisure activity that was consequently associated with better life satisfaction levels [9,11,12,13,15]. For instance, Cho and colleagues [11] examined participation in passive and active leisure activities and their effects on life satisfaction levels among older adults. The results determined that active leisure activities, such as volunteering, homemaking, and traveling, were positively associated with older adults’ life satisfaction levels. Another study investigated the effect of resistance band exercise on older adults’ life satisfaction levels. The results indicated that older adults who participated in a 12-week resistance band exercise program had significantly higher life satisfaction levels [16].

By the same token, previous studies indicated the positive impact of a special diet or dietary patterns on quality of life and life satisfaction. A systematic review study by Vajdi and Farhangi [17] evaluated 13 studies with a total of 43,445 subjects, showing that “healthy” and “Mediterranean” dietary patterns were associated with better health-related quality of life. Zaragoza-Martí and colleagues [18] evaluated the relationship between degree of life satisfaction and adherence to the Mediterranean diet among older adults. The result showed that Mediterranean diet adherence was positively related to health-related quality of life among older adults. More specifically, it showed that adherence to the Mediterranean diet was directly associated with life satisfaction levels among female older adults.

On the other hand, previous researchers have found that some lifestyles negatively affect older adults’ life satisfaction levels [1,6]. A study by Koivumaa-Honkanen and colleagues [8] showed that dissatisfaction significantly increased among men who drink heavily, as well as current smokers. Strine and colleagues [13] also found that life dissatisfaction was related to adverse health practices such as smoking, heavy drinking, and physical inactivity. Despite these findings, the direct relationship between lifestyle and life satisfaction for older adults is still ambiguous. A review by Drewnowski and Evans [14], for example, suggested that few studies have been done that relate diet quality indexes to quality of life or life satisfaction measures among older adults.

There has been little research into the effects of age difference on life satisfaction levels among older adults. Additionally, no studies have been conducted on the effects of lifestyles and health capabilities on the relationship between age difference and older adults’ life satisfaction levels. Thus, the primary purpose of this study was to determine whether older adults’ life satisfaction levels differ in terms of lifestyles and health capabilities based on the age group.

## 2. Materials and Methods

### 2.1. Study Design

The current study was a cross-sectional analysis of data drawn from the survey of the Midlife in the United Sates (MIDUS Refresher) Biomarker Project that was funded by the National Institute on Aging in the United States. The Biomarker Project was part of MIDUS 2, wherein subjects were asked to participate in a phone interview and a subsequent mail-in survey from 2012 to 2016 [19]. Three clinical research centers in the United States provided both psychosocial and biomarker assessments, which included lifestyle, health capability, and life satisfaction assessments.

### 2.2. Participants

A total of 307 adults were evaluated in the health capability assessments and self-administered questionnaire of lifestyles and life satisfaction levels, although 17 surveys had to be removed due to incompletion of the survey. Around 60 percent of the participants (*n* = 170) were male older adults in this research sample. Furthermore, the analytic sample was narrowed to include a total of 290 participants between the ages of 60 and 78 (*M*: 67.34, *SD*: 4.95) (Table 1).

### 2.3. Measurement

Lifestyles were examined by questioning participants. Participants were asked the following yes or no questions: “Do you follow a special diet?”, “Do you engage in regular exercise, or activity, of any type for 20 min or more at least three times per week?”, “Have you now or in the past used tobacco regularly?”, and “In the past month, have you had at least one drink of any alcoholic beverage such as beer, wine, wine coolers, or liquor?”.

Vital signs were measured during an overnight stay at the clinical research centers. Vitals included pulse, respiration rate, blood pressure, and temperature. Additionally, functional assessments were completed, including the following:

(1) Grip strength: recording while each subject gripped and squeezed a measurement device as hard as they could until the measurement reached a maximum;
(2) Peak flow: measuring the maximum volume of subjects’ exhalation (indicator of airway function);
(3) 50-foot timed walk: assessing the normal gait speed at which each subject walked;
(4) Chair stand: recording the time it took subjects to rise from a chair and sit down again five times while keeping arms folded across the chest.

Life satisfaction level was assessed using the Satisfaction With Life Scale (SWLS), which was introduced to provide a global measurement of a person’s life satisfaction [20]. The SWLS is a 5-item design that includes “In most ways my life is close to my ideal”, “The conditions of my life are excellent”, “I am satisfied with life”, “So far I have gotten the important things I want in life”, and “If I could live my life over, I would change almost nothing”. Each statement is completed by choosing responses on a seven-point Likert-type scale, from “Strongly disagree” (1) to “Strongly agree” (7). The score of each item was summed, yielding a range from 5 to 35, where higher scores reflect a greater life satisfaction level. The reliability of SWLS items in this study was confirmed by analysis using Cronbach’s alpha coefficient measurement (α = 0.86).

### 2.4. Analytic Strategy

Data were analyzed using the Statistical Package for the Social Sciences 20 (SPSS20) program to measure the data reliability; the descriptive proportions of age group, sex, and lifestyle; and the mean differences among age groups in terms of health capabilities and life satisfaction levels. One-way and two-way factorial analysis of variance (ANOVA) was utilized to examine the effects of age group and lifestyles on life satisfaction levels among older adults. Additionally, multiple regression analysis was used to calculate the regression coefficient to determine the linear relationships of health capabilities and age group differences on life satisfaction levels among older adults. A *p*-value of 0.05 was considered to indicate statistical significance. The homogeneity of variables and a normality test were performed prior to running the data, which confirmed that the data were appropriate to be analyzed.

## 3. Results

The group was divided manually into three age groups such that each group was equally distributed with approximately 32–35 percent of participants. The past and current lifestyles of older adults showed, in Table 1, that three-fourths of participants did not follow any special diet, while one-fourth of respondents did not engage in regular exercise or physical activity of any type for 20 min or more at least three times a week. Additionally, approximately half of the participants used tobacco regularly, now or in the past, and the consumption of any alcoholic beverage in the past month was reported by three-fourths of these older adult participants. The results of vital signs and functional assessments of the older adults were also examined in Table 1. Among the vital signs, the mean scores of pulse, respiration rate, diastolic blood pressure (DBP), and temperature were matched to the normal range. The mean score of systolic blood pressure (SBP) was higher than the normal range, but it was considered prehypertension rather than hypertension [21].

Lastly, the mean score on the SWLS in this study determined that respondents were “slightly satisfied” with their lives (*M* = 24.26). More specifically, the age group of 70 and above had the highest mean score on the SWLS (*M*: 25.05), followed by those aged 65 to 69 (*M*: 24.84) and those aged 60 to 64 (*M*: 23.00). The one-way ANOVA indicated that there was a statistically significant effect of age group on life satisfaction level. Thus, our results showed that participants of this study were more likely to be satisfied with their life with advancing age (Table 2). The main effect notwithstanding, the following pairwise comparisons did not reveal any significant group difference.

The results of the past and current lifestyles among the age groups on life satisfaction levels are shown in Table 3. The overall scores on the SWLS indicated that older adults had higher mean scores for life satisfaction levels when they engaged in exercise, while not following a special diet, smoking tobacco, and consuming alcohol did not have an impact on life satisfaction levels. More specifically, a 3 × 2 ANOVA was used to analyze the differences in life satisfaction levels by lifestyle and age group. As shown in Table 3, there were no significant differences in the interaction effect of lifestyles among age groups on the life satisfaction level. Furthermore, the main effect of special diet and tobacco consumption indicated that there was a statistically significant effect of age group on the life satisfaction level. This result also showed that the exercise group (*M*: 24.97) had a significantly higher mean life satisfaction level than did the non-exercise group (*M*: 22.03).

Multiple regression analysis was conducted to determine the impact of age group, vital signs, and functional assessment on the participants’ life satisfaction levels. Prior to analyzing the data, the variance inflation factor (VIF) test was performed to detect the multicollinearity of variables in regression analysis; the VIF index of two functional assessment variables (Grip strength—Right and Left) exceeded 5.0, resulting in their removal for multiple regression analysis. After removing these two variables, the VIF index ranged from 1.060 to 2.010, indicating appropriate usage for regression analysis. The results of the single-model regression analysis are presented in Table 4. The R-squared value showed that 26.1% of life satisfaction variance was explained by a combination of variables, including age and health capabilities. Thus, the results of the multiple regression analysis showed that respondent age has a significant influence on older adults’ life satisfaction levels. However, there were no statistical effects of vital signs or functional assessments of health capabilities on life satisfaction among older adults.

## 4. Discussion

The current study examined the direct effects of age group on older adults’ life satisfaction levels. The findings from this study show that older adults’ life satisfaction levels were significantly different based on their age group. Many studies determined that the loss of physical health capabilities causes a decrease in life satisfaction with advancing age [5,22,23]. However, the present results show the interesting fact that advancing age was positively associated to life satisfaction levels among the participating older adults. This finding supports the previous national report that older adults aged between 65 and 79 have the highest average ratings of life satisfaction level [24]. Another previous report, that the perceived level of life satisfaction increases with advancing age, is supported by the current study [25].

The current study also tested the effects of past and current lifestyles and health capabilities on life satisfaction levels among older adults. Among the lifestyle factors, only engagement in regular exercise or physical activity was significantly related to better life satisfaction levels. This result is consistent with previous studies showing that exercise, physical activity, or leisure activities are significant predictors of enhanced life satisfaction levels among older adults [11,22,26]. No significant differences were observed in the life satisfaction level with respect to special diets, tobacco use, or alcohol consumption. A previous study by Inal and colleagues [26] supports the current results, showing that health-related behaviors such as smoking and alcohol use are not statistically significant factors of life satisfaction levels among older adults.

Lastly, the present study showed the effects of health assessments and older adults’ age on life satisfaction levels. The findings in the present linear analysis revealed that respondent age was the only significant factor to affect the older adults’ life satisfaction levels. In other words, the relationship between age difference and life satisfaction levels was not mediated by health assessments, even though the explanatory power was higher in the overall linear test than in each factor’s linear test. A previous study by Chang and colleagues [27] determined the relationship between older adults’ life satisfaction levels and five areas of functional fitness status. The results indicated that some life satisfaction factors were significantly associated with some functional fitness assessment tests, but there was no overall correlation between the total fitness and the total life satisfaction levels. Another study also found that results of physical tests, including grip strength and one-legged stance tests, were not directly associated with life satisfaction levels among older women [28].

On the other hand, it might be interesting that in a previous study, the authors found that physical exercise training improved satisfaction with life, but not physical capabilities [29]. Furthermore, this study might assume that engagement in regular exercise or physical activity itself means much to older adults regardless of their current condition of vital signs or functional abilities. This meaningful behavior to attempt and to enjoy regular exercise or physical activity can influence older adults’ life satisfaction levels.

This result might match the concept of gerotranscendence, which was presented in Erikson’s extended version of life cycle theory. Gerotranscendence is the final stage of the natural process that moves toward maturation and wisdom [30]. Previous research examined the statistically significant correlation between degrees of gerotranscendence and life satisfaction levels among older adults in this stage. This stage is more cosmic and transcendent compared to the materialistic and rational vision in previous works [31,32]. Chen [23] also supports the current study’s findings of the possibility of a positive correlation between advancing age and life satisfaction because of the belief of rough to prosperous life experience and transcendence of the cohort norm on life expectancy. In this respect, older adults might transcend any circumstances they have, such as physical or social functions, and even fear of mortality, which can enable them to enhance their life satisfaction levels with normal aging.

Despite significant findings, this present study has some limitations. The main limitation of this study is the lack of demographic characteristics. This study did not provide potential covariates such as socioeconomic, social, or family status, which have an influence on older adults’ lifestyles, health capabilities, and life satisfaction levels. Previous research, for instance, indicated that older adults’ health capabilities and life satisfaction level are positively affected by family support, education, and income [33,34]. Thus, future studies would need to provide more details of demographic characteristics for a better understanding of life satisfaction levels among older adults. Another of the main limitations of this study is that the lack of a cumulative impact of lifestyles and health capability variables might disrupt the significance of the findings. Future studies should provide longitudinal data to examine the findings in greater detail.

## 5. Conclusions

The study attempted to explore the effects of advancing age, lifestyles, and health assessments on life satisfaction levels among older adults. The findings of this study showed that the relationship between advancing age and life satisfaction levels was not affected by lifestyle or health capabilities, while advancing age and engagement in exercise or physical activity significantly influenced older adults’ life satisfaction levels. This result can help explain that advancing age itself is the most crucial factor in the life satisfaction levels of older adults. Additionally, this result suggests that engagement in exercise and physical activity can facilitate older adults’ life satisfaction levels as a supplemental factor. In this respect, the current study provides an important contribution towards older adults’ life satisfaction levels through a better understanding of lifestyles, health capabilities, and advancing age. Therefore, further research is needed to optimize life satisfaction levels through appropriate programs to enhance positive lifestyles among older adults.

## Figures and Tables

**Table 1:** Research participants’ demographic characteristics and descriptive statistics of lifestyles and health capabilities.

Demographic Characteristics	*n*	%
Sex		
Female	120	41.4
Male	170	58.6
Age Group		
60–64	103	35.5
65–69	94	32.4
70–	93	32.1
**Lifestyles**	***n***	**%**
Diet		
Yes	75	25.9
No	215	74.1
Exercise		
Yes	220	75.9
No	70	24.1
Tobacco		
Yes	152	52.4
No	138	47.6
Alcohol		
Yes	216	74.5
No	74	25.5
**Health Capability**	***M***	***SD***
Vital Signs		
Pulse	70.38	11.85
Respiration Rate	16.69	1.96
Blood Pressure—Systolic	134.12	18.21
Blood Pressure—Diastolic	77.83	10.68
Temperature (Centigrade)	36.62	0.40
Functional Assessments		
Grip Strength (kg)—Right	33.49	10.83
Grip Strength (kg)—Left	31.83	10.62
Peak Flow: in L/min	450.18	126.10
50 Foot Timed Walk	13.72	2.69
Chair Stands	10.28	4.89

**Table 2:** Descriptive statistics and one-way ANOVA for life satisfaction levels on the SWLS by age group.

Life Satisfaction Level (SWLS)				*M*	*SD*
60–64				23.00	6.70
65–69				24.84	5.83
70–				25.05	5.98
Total				24.26	6.25
	**Sum of Squares**	**df**	**Mean Square**	***F***	**Sig.**
Between Groups	253.780	2	126.890	3.299	0.038
Within Groups	11,037.338	287	38.458		
Total	11,291.117	289			

**Table 3:** Descriptive statistics and two-way ANOVA for life satisfaction levels on the SWLS by lifestyle and age group.

Lifestyle		Age Group			Total (*n* = 290)	Main Effect of Lifestyle	Main Effect of Age	Interaction Effect of Exercise and Age
		60–64 (*n* = 103)	65–69 (*n* = 94)	70– (*n* = 93)				
Diet	Yes (*n* = 75)	21.74 ± 6.38	24.28 ± 6.35	25.15 ± 5.78	23.81 ± 6.24	*F*(1) = 0.808 *p* = 0.370	*F*(2) = 3.471 *p* = 0.032	*F*(2) = 0.369 *p* = 0.692
	No (*n* = 215)	23.36 ± 6.79	25.04 ± 5.67	25.02 ± 6.11	24.41 ± 6.26			
Exercise	Yes (*n* = 220)	23.67 ± 6.04	25.32 ± 5.71	25.89 ± 5.27	24.97 ± 5.74	*F*(1) = 10.689 *p* = 0.001	*F*(2) = 1.526 *p* = 0.219	*F*(2) = 0.292 *p* = 0.747
	No (*n* = 70)	21.45 ± 7.93	22.83 ± 6.07	22.19 ± 7.40	22.03 ± 7.25			
Tobacco	Yes (*n* = 152)	22.13 ± 6.43	24.15 ± 6.07	25.59 ± 5.46	23.97 ± 6.13	*F*(1) = 0.682 *p* = 0.410	*F*(2) = 3.054 *p* = 0.049	*F*(2) = 1.661 *p* = 0.192
	No (*n* = 138)	23.88 ± 6.92	25.50 ± 5.58	24.31 ± 6.64	24.57 ± 6.39			
Alcohol	Yes (*n* = 220)	23.21 ± 6.98	25.38 ± 6.00	24.89 ± 6.30	24.44 ± 6.50	*F*(1) = 0.740 *p* = 0.390	*F*(2) = 2.921 *p* = 0.055	*F*(2) = 0.843 *p* = 0.432
	No (*n* = 70)	22.38 ± 5.89	23.36 ± 5.16	25.57 ± 4.98	23.70 ± 5.47			
	Total (*n* = 290)	23.00 ± 6.70	24.84 ± 5.83	25.05 ± 5.98	24.26 ± 6.25			

**Table 4:** Unstandardized regression coefficient (***B***), standardized coefficient (***β***), and standard error (***SE***) for age and health capabilities on life satisfaction levels.

Variables	Unstandardized Coefficients		Standardized Coefficients (*β*)	*t*	*p*-Value	VIF
	*B*	*SE*				
(Constant)	−14.780	35.112		−0.421	0.674	
Age group	0.256	0.077	0.202	3.341	0.000	1.087
Vital Signs						
Purse	−0.009	0.033	−0.018	−0.292	0.771	1.121
Respiration Rate	−0.379	0.192	−0.118	−1.970	0.050	1.032
SBP	−0.029	0.028	−0.084	−1.054	0.293	1.910
DBP	0.017	0.048	0.029	0.350	0.727	2.010
Temperature (°C)	0.789	0.927	0.050	0.851	0.396	1.026
Functional Assessments						
Peak Flow: in L/min	0.006	0.004	0.128	1.804	0.072	1.504
50 Foot Timed Walk	0.014	0.162	0.006	0.086	0.932	1.437
Chair Stands	−0.0133	0.086	−0.026	−0.387	0.699	1.349
*R*^2^ = 0.261, *F* (9, 277) = 2.247, *p* = 0.019						

## Data Availability

The datasets generated during and/or analyzed during the current study are available at https://www.icpsr.umich.edu/web/ICPSR/studies/36901 (accessed on 24 March 2023).
